# 6,7-Di­hydro-5*H*-pyrrolo­[1,2-*a*]imidazole

**DOI:** 10.1107/S2414314620006811

**Published:** 2020-05-29

**Authors:** Oscar Morales-Collazo, Vincent M. Lynch, Joan F. Brennecke

**Affiliations:** a McKetta Department of Chemical Engineering, University of Texas at Austin, Austin, Texas 78712, USA; b Department of Chemistry, University of Texas at Austin, Austin, Texas 78712, USA; Vienna University of Technology, Austria

**Keywords:** crystal structure, imidazole derivative, ring puckering analysis

## Abstract

The crystal structure of 6,7-di­hydro-5*H*-pyrrolo­[1,2-*a*]imidazole, C_6_H_8_N_2_, at 100 K has monoclinic (*P*2_1_/*n*) symmetry. The mol­ecule adopts an envelope conformation of the pyrrolidine ring, which might help for the relief torsion tension. The crystal cohesion is achieved by C—H⋯N hydrogen bonds. Inter­estingly, this fused ring system provides protection of the α-C atom (attached to the non-bridging N atom of the imidazole ring), which provides stability that is of inter­est with respect to electrochemical properties as electrolytes for fuel cells and batteries, and electrodeposition.

## Abstract 2

In the title compound, the pyrrolidine ring fused to the imidazole ring has an envelope conformation.

## Graphical abstract

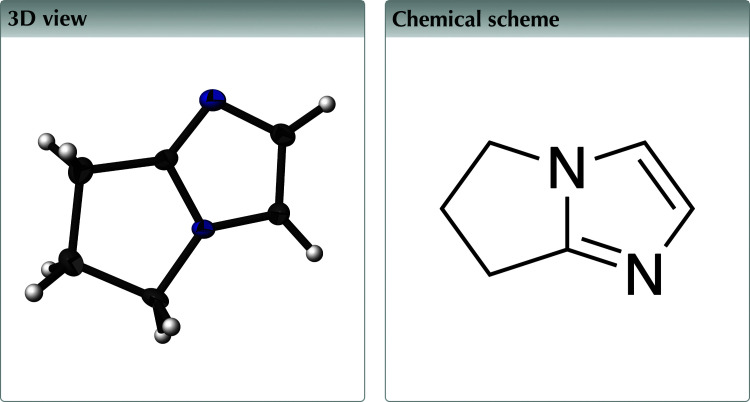

## Structure description

**Figure 1:**
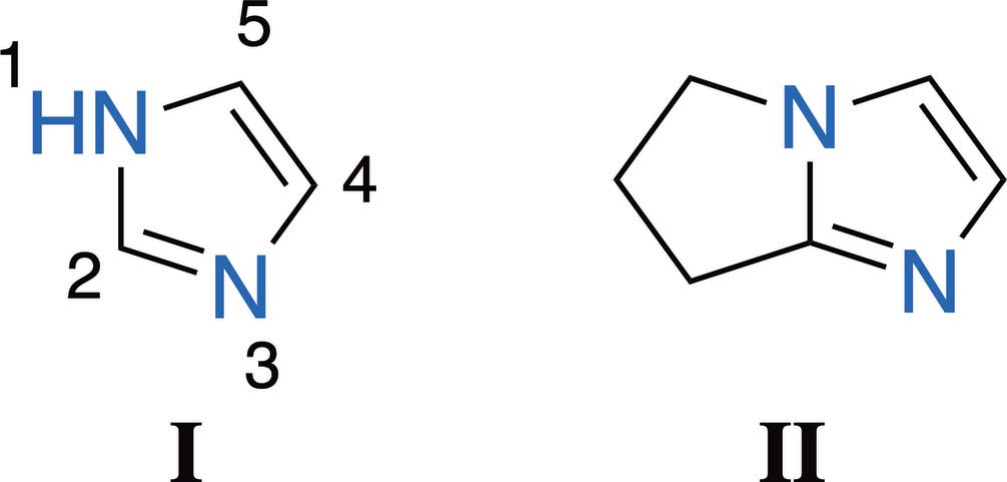
Schematic representation of imidazole (**I**) with atom numbering and of the title derivative (**II**).

Ionic liquids have emerged as a promising area in material science because of their tunable properties, allowing them to be used in a wide range of application such as: carbon dioxide capture, fuel cells, nanoparticle stabilization, and many more (Song *et al.*, 2019 ▸; Huang *et al.*, 2017 ▸; Wang *et al.*, 2017 ▸). In this context, our group has been working on the synthesis of new cation moieties for ionic liquid designs. From the different chemical entities employed in ionic liquids, imidazolium derivatives are widely used in the field due to their versatility and relatively high stability. Imidazolium ionic liquid research is dominated by fluorine containing anions (Xue *et al.*, 2006 ▸). Thus, we intended to explore imidazole derivatives (**II**) at position 2 of the principal structural component, imidazole (**I**), in order to decrease its reactivity (Fig. 1 ▸).

To understand how cations and anions inter­act in ionic liquids, characterization of the starting materials is important. Towards that end, the crystal structure of 6,7-di­hydro-5*H*-pyrrolo­[1,2-*a*]imidazole (**II**) is presented in order to characterize and establish a structure stability relationship of cyclic imidazole derivative families for imidazolium ionic liquid research applications.

**Figure 2:**
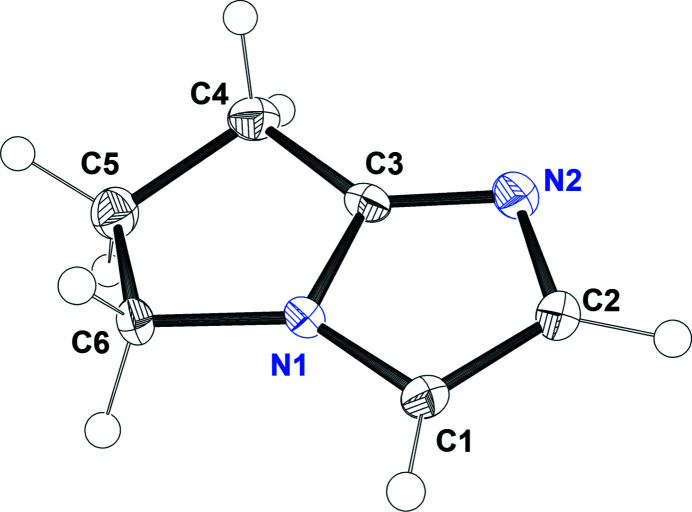
The mol­ecular structure of (**II**) showing the atom-labeling scheme. Displacement ellipsoids are scaled to the 50% probability level.

The mol­ecular structure of imidazole derivative (**II**) is displayed in Fig. 2 ▸. We initially envisioned that the electronic and steric effects would be similar to a pyrrole fused to the imidazole moiety and, thus, provide comparison to pyrrolidine below. In order to put into perspective the results found in the mol­ecular and crystal structure of (**II**), we compare the fused imidazole moiety of (**II**) with the imidazole crystal structure. There is no significant difference in bond length of the imidazole moiety of (**II**) compared to the imidazole crystal structure (McMullan *et al.*, 1979 ▸). However, the C2—N2 (N3—C4 in imidazole; McMullan *et al.*, 1979 ▸) bond length of (**II**) is larger than the same bond found in the imidazole crystal structure [1.390 (2) *vs*1.375 (1) Å]. This bond-length difference might be due to the new substituted imidazole ring system, which can help justify the chemical shifts observed in the ^1^H-NMR spectrum between (**II**) and (**I**) of those hydrogen atoms in C1 and C2 of (**II**), based on the inductive effects of the substituents. On the other hand, we also compare the pyrrolidine fused ring to the pyrrolidine crystal structure (Dziubek & Katrusiak, 2011 ▸). We found that the major bond length difference occurs between bonds N1—C3 and C3—C4 [1.353 (2) *vs* 1.457 (2) Å and 1.492 (2) *vs* 1.528 (2) Å], respectively; only the shortest bond length of pyrrolidine was used due to its symmetry). The C5—C6 bond length was found to be 1.543 (2) Å, which is slightly larger than the one found for pyrrolidine [1.528 (2) Å]. These differences in bond length could be attributed to the new *sp* ^2^ carbon atom (C3) of (**II**), which also might be responsible for the differences in bond angles in (**II**). For example, angles N1—C3—C4 and C6—N1—C3 within the fused ring system are much larger compared to those of the pyrrolidine structure [111.1 (1) *vs* 107.2 (1) and 113.99 (9) *vs* 103.37 (1) Å, respectively], but angle N1—C6—C5 [102.10 (9) *vs* 107.05 (1) Å] becomes smaller. These angle differences, despite being small, can help relieve the ring’s stress. Finally, it is important to point out that N1 in pyrrolidine is out of plane (envelope conformation) in order to reduce lone-pair inter­actions, but in (**II**), this envelope conformation is adopted by C5 as the flap atom where C5 is 0.317 (2) Å out of the plane of the remaining four atoms. Also, the imidazole ring and the planar part of the pyrrolidine ring make a dihedral angle of 3.85 (9)°. By N1 becoming part of the plane, its lone pairs could add new repulsion inter­actions, suggesting why the ring has to adapt to this conformation to avoid repulsions.

**Figure 3:**
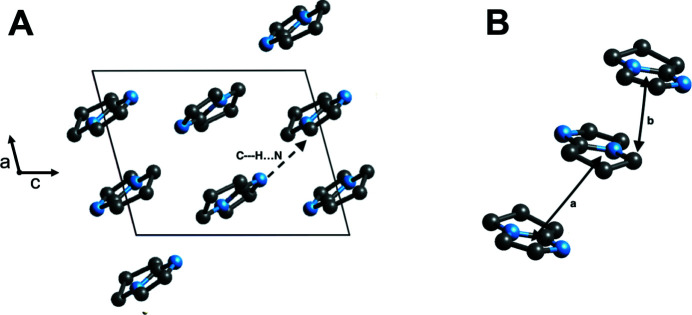
Packing diagram for (**II**) projected along the *b* axis (A), and the inter­molecular arrangement and distance of (**II**) found in the crystal structure (B) (a shows the centroid-to-centroid distance, b the centroid-to-atom distance). Hydrogen atoms were omitted for clarity.

A look into possible inter­molecular hydrogen-bonding inter­actions of (**II**), we found a value of 3.37 (3) Å between C1⋯N2, indicative of a weak hydrogen bond (Fig. 3 ▸, Table 1 ▸) that leads to the formation of supra­molecular chains extending parallel to [101]. Nevertheless, we believe that the major inter­molecular force contribution to the stabilization of the crystal structure is by aliphatic C—H⋯π inter­actions. We observed how C6 (non-aromatic ring atom) inter­acts with C2^i^, N2^i^, and C3^i^ [distances are 3.672 (3), 3.692 (4), and 3.620 (3), respectively; symmetry code: (i) −*x* + 1, −*y* + 2, −*z*). Analyzing the possibility of any π–π inter­actions we determine that due to the reciprocal stacking of the mol­ecules and the offset distance of the aromatic centroids (4.487 Å), we suggest that the possibility of a π–π inter­action between the mol­ecules is not found. We conclude that C—H⋯π inter­actions, although weak compared to a conventional hydrogen bond, could serve together with other inter­molecular forces to impose directionality and order through the crystal lattice. Previously, C—H⋯π inter­actions have been proven to show stability in crystal structures. In fact, it has been suggested that aliphatic–aromatic inter­actions could play a greater stabilization role than aromatic–aromatic inter­actions (Ninković *et al.*, 2016 ▸; Carmona-Negrón *et al.*, 2016 ▸). Fig. 3 ▸ shows the packing diagram of (**II**).

## Synthesis and crystallization

The compound was synthesized following a literature procedure with a modification (Kan *et al.*, 2007 ▸). Hydrogen chloride was bubbled into a solution of 4-chloro­butyro­nitrile (10 g, 274 mmol) and methanol (11.65 ml, 288 mmol) in ether (135 ml). The solution was treated with hydrogen chloride at room temperature until saturated. After 24 h of reaction, the white precipitate was washed with ether and dried under vacuum to afford the imidate. ^1^H NMR (400 MHz, CDCl_3_) δ 12.51 (*s*, 1H), 11.60 (*s*, 1H), 4.26 (*s*, 3H), 3.57 (*t*, *J* = 6.1 Hz, 2H), 2.92 (*t*, *J* = 6.6 Hz, 2H), 2.19 (*q*, *J* = 6.3 Hz, 2H).

To a solution of the imidate (34 g, 198 mmol) in di­chloro­methane (200 ml), amino­acetaldehyde (20.78 g, 198 mmol) and tri­ethyl­amine (60 g, 593 mmol) were added and heated to 333 K for 2 h to afford the amidine inter­mediary, which was dried under vacuum. The amidine was stirred in formic acid at 353 K for 20 h. Solid sodium bicarbonate was added to the solution to raise the pH to 10. The solution was extracted with di­chloro­methane (3 × 100 ml) and dried over anhydrous Na_2_SO_4_. Filtration and evaporation under reduced pressure was followed by sublimation to afford a crystalline solid (12.7 g, 60% yield in two steps); m.p. 338 K; ^1^H NMR (400 MHz, DMSO-*d*_6_) δ 7.02 (*d*, *J* = 1.3 Hz, 1H), 6.85 (*d*, *J* = 1.3 Hz, 1H), 3.89 (*dd*, *J* = 7.6, 6.6 Hz, 2H), 2.66 (*m*, 2H), 2.44 (*m*, 2H).

Crystals of the title compound grew as very large, colorless prisms by slow sublimation at 313 K and 1.5 mbar. The crystal under investigation was cut from a larger crystal.

## Refinement

Crystal data, data collection and structure refinement details are summarized in Table 2 ▸.

## Supplementary Material

Crystal structure: contains datablock(s) . DOI: 10.1107/S2414314620006811/wm4129sup1.cif

Structure factors: contains datablock(s) I. DOI: 10.1107/S2414314620006811/wm4129Isup2.hkl

Supporting information file. DOI: 10.1107/S2414314620006811/wm4129Isup3.cml

CCDC reference: 1912032

Additional supporting information:  crystallographic information; 3D view; checkCIF report

## Figures and Tables

**Table 1:** Hydrogen-bond geometry (Å, °)

*D*—H⋯*A*	*D*—H	H⋯*A*	*D*⋯*A*	*D*—H⋯*A*
C1—H1⋯N2	0.95 (1)	2.52 (1)	3.73 (3)	150 (1)

**Table 2:** Experimental details

Crystal data	
Chemical formula	C_6_H_8_N_2_
*M*_r_	108.14
Crystal system, space group	Monoclinic, *P*2_1_/*n*
Temperature (K)	100
*a*, *b*, *c* (Å)	7.908 (7), 7.441 (8), 9.880 (9)
β (°)	104.91 (3)
*V* (Å^3^)	561.8 (9)
*Z*	4
Radiation type	Mo *K*α
μ (mm^−1^)	0.08
Crystal size (mm)	0.31 × 0.19 × 0.16

Data collection	
Diffractometer	Rigaku AFC-12 with Saturn 724+ CCD
Absorption correction	Multi-scan (*ABSCOR*; Higashi, 2001 ▸)
*T*_min_, *T*_max_	0.730, 1.00
No. of measured, independent and observed [*I* > 2σ(*I*)] reflections	2575, 1279, 1028
*R*_int_	0.036
(sin θ/λ)_max_ (Å^−1^)	0.649

Refinement	
*R*[*F* ^2^ > 2σ(*F* ^2^)], *wR*(*F* ^2^), *S*	0.042, 0.123, 1.05
No. of reflections	1279
No. of parameters	105
H-atom treatment	All H-atom parameters refined
Δρ_max_, Δρ_min_ (e Å^−3^)	0.28, −0.22

Computer programs: *CrystalClear* (Rigaku, 2008 ▸), *SHELXT* (Sheldrick, 2015*a* ▸), *SHELXL* (Sheldrick, 2015*b* ▸), *XP* in *SHELXTL/PC* (Sheldrick, 2008 ▸), *Mercury* Macrae *et al.*, 2020 ▸) and *publCIF* (Westrip, 2010 ▸).

## full crystallographic data

### Crystal data

**Table:**

C_6_H_8_N_2_	*F*(000) = 232
*M**_r_* = 108.14	*D*_x_ = 1.279 Mg m^−^^3^
Monoclinic, *P*2_1_/*n*	Mo *K*α radiation, λ = 0.71073 Å
*a* = 7.908 (7) Å	Cell parameters from 1451 reflections
*b* = 7.441 (8) Å	θ = 2.7–27.5°
*c* = 9.880 (9) Å	µ = 0.08 mm^−^^1^
β = 104.91 (3)°	*T* = 100 K
*V* = 561.8 (9) Å^3^	Prism, colorless
*Z* = 4	0.31 × 0.19 × 0.16 mm

### Data collection

**Table:**

Rigaku AFC-12 with Saturn 724+ CCD diffractometer	1028 reflections with *I* > 2σ(*I*)
Radiation source: sealed fine focus tube	*R*_int_ = 0.036
ω–scans	θ_max_ = 27.5°, θ_min_ = 3.0°
Absorption correction: multi-scan (*ABSCOR*; Higashi, 2001)	*h* = −6→10
*T*_min_ = 0.730, *T*_max_ = 1.00	*k* = −6→9
2575 measured reflections	*l* = −12→12
1279 independent reflections	

### Refinement

**Table:**

Refinement on *F*^2^	0 restraints
Least-squares matrix: full	Hydrogen site location: difference Fourier map
*R*[*F*^2^ > 2σ(*F*^2^)] = 0.042	All H-atom parameters refined
*wR*(*F*^2^) = 0.123	*w* = 1/[σ^2^(*F*_o_^2^) + (0.0797*P*)^2^] where *P* = (*F*_o_^2^ + 2*F*_c_^2^)/3
*S* = 1.05	(Δ/σ)_max_ < 0.001
1279 reflections	Δρ_max_ = 0.28 e Å^−^^3^
105 parameters	Δρ_min_ = −0.22 e Å^−^^3^

### Special details

**Table:**

Geometry. All esds (except the esd in the dihedral angle between two l.s. planes) are estimated using the full covariance matrix. The cell esds are taken into account individually in the estimation of esds in distances, angles and torsion angles; correlations between esds in cell parameters are only used when they are defined by crystal symmetry. An approximate (isotropic) treatment of cell esds is used for estimating esds involving l.s. planes.

### Fractional atomic coordinates and isotropic or equivalent isotropic displacement parameters (Å^2^)

**Table:**

	*x*	*y*	*z*	*U*_iso_*/*U*_eq_	
C1	0.35368 (15)	0.73358 (16)	0.04868 (12)	0.0174 (3)	
C2	0.25317 (16)	0.68855 (17)	−0.08171 (12)	0.0197 (3)	
C3	0.20769 (14)	0.96844 (16)	−0.05783 (11)	0.0156 (3)	
C4	0.17443 (17)	1.16552 (17)	−0.05457 (13)	0.0209 (3)	
C5	0.26013 (17)	1.21318 (18)	0.10150 (13)	0.0219 (3)	
C6	0.38632 (16)	1.05792 (16)	0.16201 (12)	0.0183 (3)	
N1	0.32126 (12)	0.91323 (13)	0.06209 (9)	0.0150 (3)	
N2	0.16179 (13)	0.83657 (14)	−0.14934 (10)	0.0194 (3)	
H1	0.4341 (18)	0.6695 (18)	0.1200 (15)	0.024 (4)*	
H2	0.2433 (17)	0.5700 (17)	−0.1276 (14)	0.023 (4)*	
H4A	0.2310 (17)	1.2310 (17)	−0.1188 (14)	0.023 (4)*	
H4B	0.0496 (19)	1.1952 (19)	−0.0828 (15)	0.029 (4)*	
H5A	0.3296 (19)	1.333 (2)	0.1160 (16)	0.032 (4)*	
H5B	0.167 (2)	1.2194 (19)	0.1527 (15)	0.035 (4)*	
H6A	0.5118 (17)	1.0873 (18)	0.1648 (13)	0.022 (3)*	
H6B	0.3805 (18)	1.018 (2)	0.2585 (15)	0.036 (4)*	

### Atomic displacement parameters (Å^2^)

**Table:**

	*U*^11^	*U*^22^	*U*^33^	*U*^12^	*U*^13^	*U*^23^
C1	0.0210 (6)	0.0136 (6)	0.0166 (6)	−0.0006 (5)	0.0029 (5)	0.0022 (5)
C2	0.0246 (6)	0.0159 (7)	0.0177 (6)	−0.0034 (5)	0.0037 (5)	−0.0014 (5)
C3	0.0149 (5)	0.0193 (6)	0.0123 (6)	0.0003 (4)	0.0029 (4)	0.0023 (4)
C4	0.0232 (7)	0.0201 (7)	0.0199 (6)	0.0025 (5)	0.0067 (5)	0.0036 (5)
C5	0.0255 (7)	0.0192 (7)	0.0224 (6)	0.0004 (5)	0.0087 (5)	−0.0032 (5)
C6	0.0238 (6)	0.0178 (6)	0.0128 (6)	−0.0054 (5)	0.0038 (4)	−0.0037 (4)
N1	0.0174 (5)	0.0158 (6)	0.0111 (5)	−0.0014 (4)	0.0021 (4)	−0.0001 (4)
N2	0.0207 (5)	0.0210 (6)	0.0155 (5)	−0.0017 (4)	0.0025 (4)	−0.0004 (4)

### Geometric parameters (Å, º)

**Table:**

C1—C2	1.3700 (19)	C4—H4A	0.993 (14)
C1—N1	1.374 (2)	C4—H4B	0.980 (15)
C1—H1	0.948 (14)	C5—C6	1.543 (2)
C2—N2	1.3905 (18)	C5—H5A	1.038 (15)
C2—H2	0.986 (13)	C5—H5B	0.994 (15)
C3—N2	1.3203 (18)	C6—N1	1.4619 (17)
C3—N1	1.3533 (17)	C6—H6A	1.010 (13)
C3—C4	1.492 (2)	C6—H6B	1.011 (14)
C4—C5	1.557 (2)		
			
C2—C1—N1	104.54 (10)	C6—C5—H5A	108.9 (8)
C2—C1—H1	133.8 (8)	C4—C5—H5A	114.4 (8)
N1—C1—H1	121.6 (8)	C6—C5—H5B	109.0 (8)
C1—C2—N2	111.23 (12)	C4—C5—H5B	108.9 (8)
C1—C2—H2	127.5 (8)	H5A—C5—H5B	108.9 (12)
N2—C2—H2	121.2 (8)	N1—C6—C5	102.10 (11)
N2—C3—N1	112.18 (12)	N1—C6—H6A	110.5 (7)
N2—C3—C4	136.59 (11)	C5—C6—H6A	112.4 (8)
N1—C3—C4	111.13 (10)	N1—C6—H6B	109.0 (9)
C3—C4—C5	102.18 (10)	C5—C6—H6B	113.8 (9)
C3—C4—H4A	111.0 (8)	H6A—C6—H6B	108.7 (10)
C5—C4—H4A	111.6 (8)	C3—N1—C1	108.01 (10)
C3—C4—H4B	112.7 (8)	C3—N1—C6	113.99 (11)
C5—C4—H4B	112.4 (8)	C1—N1—C6	137.70 (10)
H4A—C4—H4B	107.1 (11)	C3—N2—C2	104.03 (12)
C6—C5—C4	106.64 (11)		
			
N1—C3—C4—C5	−10.83 (13)	C4—C3—N1—C6	−1.63 (13)
C3—C4—C5—C6	18.49 (13)	C5—C6—N1—C3	13.39 (12)
C4—C5—C6—N1	−19.28 (13)		

### Hydrogen-bond geometry (Å, º)

**Table:**

*D*—H···*A*	*D*—H	H···*A*	*D*···*A*	*D*—H···*A*
C1—H1···N2	0.95 (1)	2.52 (1)	3.73 (3)	150 (1)

### Selective Supramolecular interaction of II

**Table:**

D···A	Distance
C6-N2	3.692 (4)
C6-C2	3.672 (3)
C6-C3	3.620 (3)
